# Personal

**Published:** 1906-06

**Authors:** 


### PERSONAL.

Dr. Augustus Grote Pohlman, formerly of Buffalo, and a medical graduate of the University of Buffalo, 1900, has been appointed associate professor of anatomy in Indiana University, at Bloomington. Dr. Pohlman, who is a son of Dr. Julius Pohlman, the ophthalmologist of Buffalo, has been assistant professor of anatomy in the Indiana University for two years past, hence his promotion will be gratifying to his many friends, as well as the friends of his father, residing in this city.

---

Dr. J. B. S. Holmes, of Atlanta, has been chosen president of the Georgia State Medical Examining Board.

---

Dr. William Warren Potter, of Buffalo, was appointed by Governor Higgins a delegate to represent the State of New York at a conference of the council on medical education of the American Medical Association, held at Chicago, May 12, 1906.

---

Dr. Walter B. Chase, of Brooklyn, has removed from 1045 Prospect Place to 936 St. Mark's Avenue.

---

Dr. George F. Cott, of Buffalo, whose interesting and instructive article "A Series of Tracheotomies," appears elsewhere in this issue of the Journal, has removed his office from 85 North Pearl Street to 1248 Main Street. Hours 9 - 1. Both telephones.

---

Dr. William J. O'Donnell, of Buffalo, will sail for Europe early in June, where he will study for several months in the principal medical educational centers.

---

Professor A. B. Macallum, of Toronto University, has been elected a member of the Royal Society of London. In 1884 he became a fellow of Toronto University, lecturing in physiology; in 1891 he was appointed professor of physiology in the medical faculty, and in 1892 he took the professorship of physiology in the arts faculty. He has made physiology a life study and has applied himself more particularly to that phase of the science which deals with cell life. Here he has thrown much light upon the subject as a result of his observations, and has gained wide celebrity in his department of effort.—*The Tribune.*

---

Dr. W. J. Goodhue, medical superintendent of the leper settlement at Molokai, has written a letter to a friend in Toronto, saying that he has discovered the germ of leprosy in the mosquito and in vermin. Dr. Goodhue was born at Habaskaville, Quebec, October 8, 1869, and is a personal friend of Sir Wilfrid Laurier.—*The Tribune.*

---

Dr. N. G. Russell, of Buffalo, sailed for Europe May 26, 1906, to be gone several months for the purpose of post-graduate study.

Dr. N. L. Burnham, of Buffalo, has gone to Europe to engage in clinical observations for a number of months.

---

Dr. Eugene A. Smith, of Buffalo, is on the way to Europe for the purpose of recreation and to visit some of the most noted surgical clinics in England and on the continent.

---

Dr. Harry R. Trick, of Buffalo, has removed from 482 Delaware Avenue to 1125 Main Street, corner of Dodge.

---

Dr. Charles D. Hauser, of Youngstown, O., sailed for Europe during the latter days of May to engage in study in some of the famous clinics of the old country.

---

Dr. Edward N. Brush, Superintendent of the Sheppard and Enoch Pratt Hospital, Baltimore, an alumnus of the University of Buffalo, 1874, delivered the address to the class of 1906, at the recent annual commencement of that institution. We expect to publish it in a future issue of the Journal.

---

Dr. Jacob E. Helwig, of North Tonawanda, has been appointed a member of the board of public works of that city.

